# A flexible power electronic converter system with rapid control prototyping for research and teaching

**DOI:** 10.1016/j.ohx.2023.e00411

**Published:** 2023-03-04

**Authors:** Tommaso Caldognetto, Andrea Petucco, Andrea Lauri, Paolo Mattavelli

**Affiliations:** Department of Management and Engineering, University of Padova, Vicenza, Italy

**Keywords:** Power electronics, Inverters, Rapid control prototyping, Experimental setups

## Abstract

A flexible power electronic converter embedding a rapid control prototyping platform suitable to be applied in research test setups and teaching laboratories is proposed and described in this paper. The electronic system is composed of three subsystems, namely, *i*) three half-bridge power boards, *ii*) a dc-link capacitor bank with a half-bridge power module for active dc-link control, *iii*) an interfacing board, called motherboard, to couple the power modules with a control unit, *iv*) a digital control unit with rapid control prototyping functionalities for controlling power electronic circuits. Power modules integrate sensors with related conditioning circuits, driving circuits for power switches, and protection circuits. Conversion circuits exploit GaN electronic switches for optimal performance. The architecture and implementation of the system are described in detail in this manuscript. Main applications are in the implementation of conversion circuits for supplying arbitrary ac or dc voltages or currents, testing of new control algorithms for power electronic converters, testing of systems of electronic converters in, for example, smart nanogrids or renewable energy applications, training of undergraduate and graduate students.

## Graphical abstract

**Figure:**
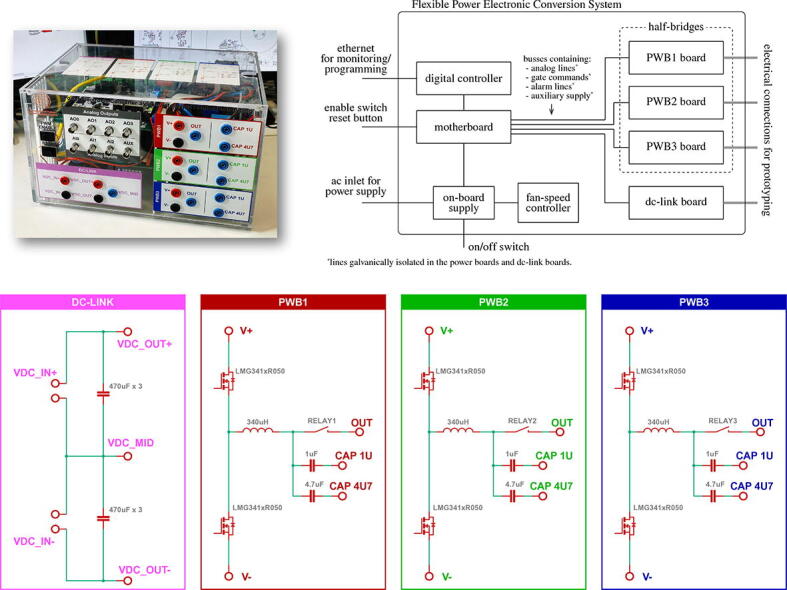

**Specifications table:**

**Table:** 

**Hardware Name**	Power Electronic Converter System with Rapid Control Prototyping

**Subject Area**	• Electrical and Electronic Engineering • Educational Tools and Open Source Alternatives to Existing Infrastructure • General
**Hardware Type**	• Electronics engineering and computer science • Electrical engineering
**Closest Commercial Analog**	No equivalent commercial analog is available. Some similar solutions can be found in [1], [2] or [3]. These are considered in the introduction of the manuscript.
**Open Source License**	Open source license CERN-OHL-P (latest version).
**Cost of Hardware**	Approximate cost of hardware: 5 kEur.
**Source File Repository**	Available in the article and at the on-line repository [4].

## 1 Hardware in context

Power electronics circuits for energy conversion are a crucial technology for countless classical and modern applications and are the beating heart of the ongoing electrification of the energy sector [5]. According to [6], more than 70% of all electrical energy today is processed by power electronics, and this will increase in the coming decades. Such an increase is further accelerated by the decarbonization pledges taken by many countries and the urgency, due to multiple reasons, of transitioning from fossil fuels to alternative energies. In any case, the path toward decarbonization and a fossil-fuel-free energy sector appears paved by power electronics solutions [7].

System-level applications and controllers are a substantial part of power electronics in industry and research. The share of published papers having their main research contribution in controls is substantial in generic power electronics journals (e.g., 25% in 2019 according to [8]), with an increasing number of system-level applications. However, the prototyping of setups for testing controls or system-level applications is typically a delicate task, because the design and implementation of practical power circuits require dedicated know-how, tools, and resources [9], [10]. Various challenges arise at this point: *i*) design of a suitable system, including the design of the analog circuits, power circuits, and components selection; *ii*) physical implementation, requiring specific know-how and laboratory equipment; *iii*) ensure a flexible design to make the solution adaptable to various configurations and control techniques to be implemented and tested.

Similar considerations apply when considering the training of students in university courses related to power electronics, mechatronics, and electrical engineering [11]. In this case, an additional tradeoff should be accommodated: on the one hand, students should be exposed to state-of-the-art, meaningful setups, on the other hand, the effort should focus on the specific aspect under study. A solution often applied is the use of hardware-in-the-loop setups, as shown, for example, in [12], which present several limitations related to considering a hardware emulation instead of a real hardware realization.

Then, the proposed hardware is a general-purpose power electronic system that can be used for the conversion of electrical power in research or teaching laboratories of power electronics, digital control in power electronics, and power electronics for renewable energies. The system is composed of three half-bridges of electronic switches rated 400V-dc, $12\;{\text{A}}_{\mathrm{rms}}$, with integrated output *LC*-filter, a dc-link board constituted of the series connection of two banks of electrolytic capacitors, a dedicated half-bridge of electronic switches if active control of the split capacitor bank voltage is needed, and all the current and voltage sensors required for the monitoring and control of the electronic conversion system. The system embeds a digital rapid control prototyping (RCP) board to control the system, allowing full access to the hardware.

The electronic switches are based on gallium nitride (GaN) field effect transistors rated 600V-dc, 50mΩ, by Texas Instruments (LMG341xR050), with integrated over-temperature and overcurrent protection. Wide-bandwidth analog circuits are employed for accurate measurements of voltages and currents. A protection hierarchy, including both hardware and software provisions, is implemented to ensure safe operation during the validation of research outcomes and operation with trainees (e.g., undergraduate students). Access to the electrical terminals of the power conversion boards is allowed by on-board connectors and related banana connectors on the front-panel of the system. On-board and banana connectors allow to arbitrarily interconnect the internal power conversion circuits to implement ad hoc power conversion topologies for research or teaching.

A few commercial alternative solutions may be found, like, for example, [1], [2] or [3]. With respect to these, the designed open-source hardware described herein presents switches based on GaN transistors and is devised for being connected to an advanced rapid control prototyping controller. In addition, it presents lower costs and a flexible and modular structure that can be easily adapted to the conversion circuit to be tested. Research papers also report some alternatives. In [13] a reconfigurable rapid prototyping platform is presented for power electronic circuits and systems, with more limited voltage and power ratings, which may impede the implementation of realistic validation scenarios for research. A similar approach is shown in [14], showing that Matlab/Simulink can be exploited for coding embedded digital controllers. This last approach allows a rapid prototyping of the digital control algorithm, and it is exploited in this paper too for the firmware programming part. Another example is given in [15], [16], where a set of plug-and-play power electronic boards is developed for teaching purposes, exploiting Matlab/Simulink embedded coder to program a microcontroller. The boards allow students to study circuit-level characteristics of electronic converters and easily implement fundamental control schemes (e.g., voltage and current regulation loops). From a more system-level perspective, the solution presented herein proposes a closed-box flexible power electronic converter system that allows the rapid prototyping of common conversion structures and testing of basic and advanced control algorithms for teaching and research applications. The emulation of validation hardware setups is also possible, giving results valuable to validate the effectiveness of control principles. Examples are described in [17], [12]. Disadvantages of such approaches consist in lacking all the real-word non-idealities, non-linearities, and relevant limitations that a real hardware realization actually presents.

The solution proposed herein aims at overcoming such limitations, presenting a flexible power electronic system implemented on hardware. In particular, a rapid prototyping system integrating state-of-the-art power electronics hardware and digital control platform allowing rapid control prototyping is considered and shown herein. The features and the relevant implementation details of the proposal are discussed in the following sections.

## 2 Hardware description

The designed hardware is devised to fulfill the common need of power electronics laboratories of disposing of a flexible hardware system coupled to a high-performance digital controller to allow *i*) the experimental validation of novel control methods proposed to the scientific community, as well as *ii*) the training of students using a platform that is complete, realistic, and safe. Based on this need, the proposed design presents the following features:

• suitable voltage, current, and power for testing applications of practical interest,
• multiple levels of protection to ensure safety for the user (e.g., students) and minimize component failures during laboratory experiments,
• use of wide-bandgap devices to achieve a compact solution with good conversion efficiency,
• use of modern high-performance electronic devices to achieve high-performance during research-related activities and, during training sessions, an intriguing up-to-date platform for students,
• a high-end digital controller including both microprocessor and field programmable gate arrays (FPGA) for full control flexibility and computation capability,
• compatibility with rapid control prototyping paradigm,
• wide bandwidth and isolated sensors for facilitating the implementation of high-performance digital control techniques,
• hardware modules galvanically isolated for the control board,
• stand-alone solution not requiring additional external components/power-supplies, protection, electro-magnetic filtering, etc.,
• modular and open hardware structure allowing updates and expansions; open hardware is important to be fully aware of the hardware characteristics (e.g., transfer functions, gains, linearity), whose knowledge is crucial for designing high-performance digital controllers and, in general, for optimal control design.

**Fig. 1:**
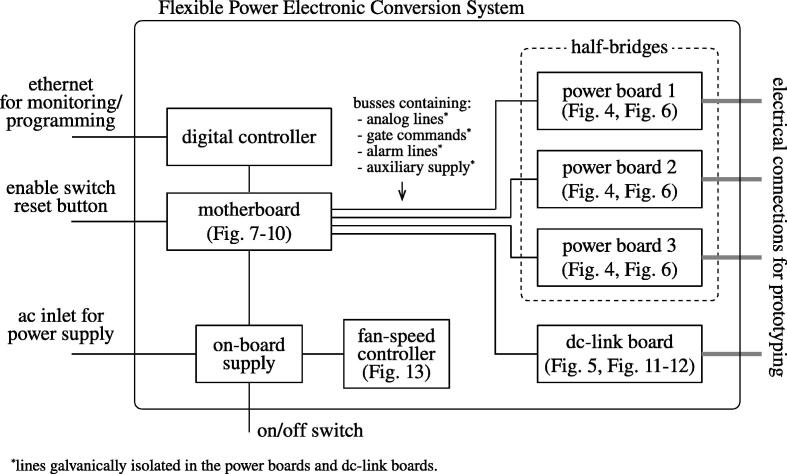
Block scheme of the proposed flexible power electronic converter system with rapid control prototyping.

Fig. 1 reports the main constituting components of the proposed system.

### 2.1 Half-bridge section with power boards

**Fig. 4:**
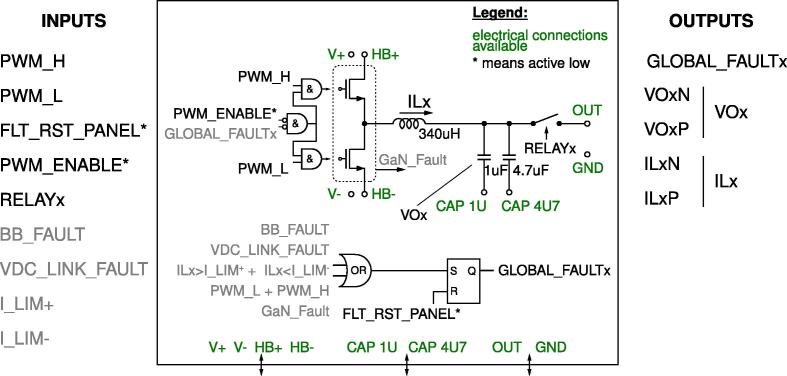
Schematic representation of the half-bridge power board. On the left, the input signals of the board, on the right, the output signals generated by the board. On the bottom, colored in green, the power lines made available at dedicated connectors. The complete schematic of the board is provided in Fig. 6.

**Fig. 6:**
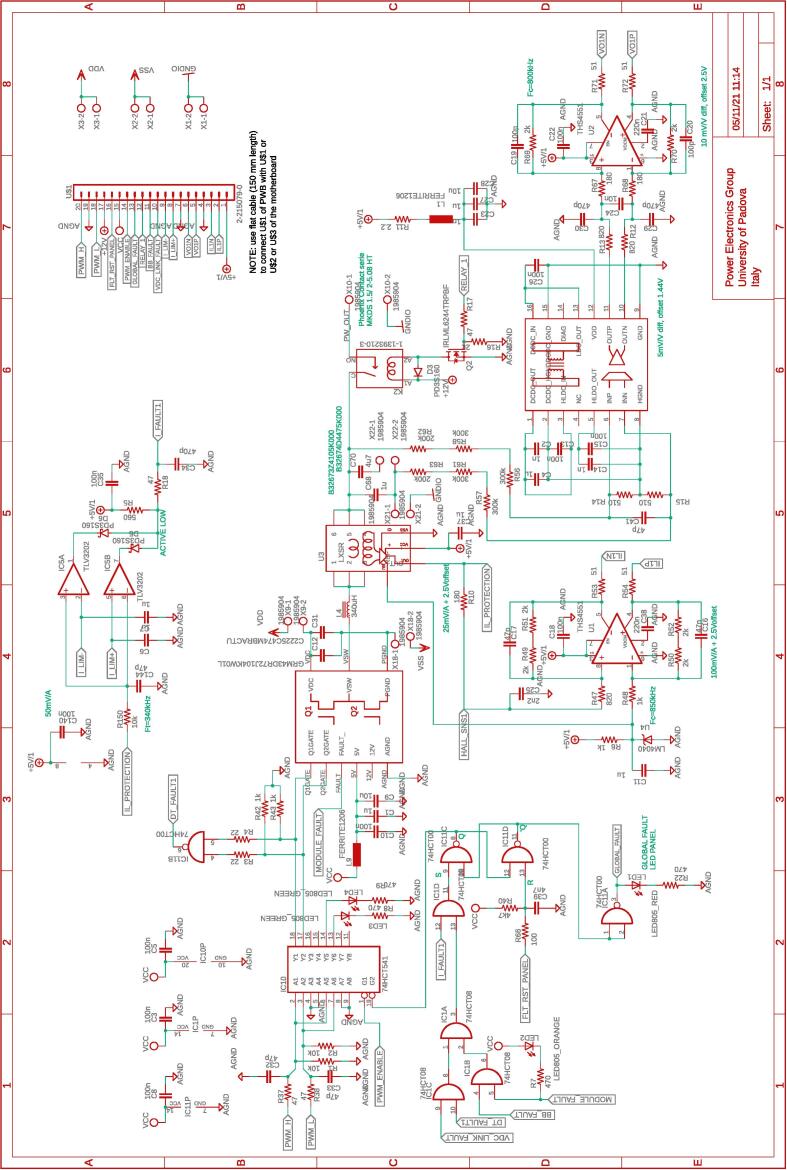
Power board implementing a half-bridge based on GaN modules and including measurement and isolation circuits.

The half-bridge section is the heart of the rapid prototyping system hardware. This section comprises three independent and galvanically isolated power boards (PWB). Each power board embeds a Texas Instrument LMG3411EVM-018 half-bridge module based on two LMG341xR050 devices. Each device integrates a GaN FET switch, a gate-driver, and overcurrent and over-temperature protections. Then an *LC* output filter is present, constituted of a 340-μH inductor, a 1-μF capacitor, and a 4.7-μF capacitor. The capacitors can be connected as needed, while the output voltage measurement is performed across the 1-μF capacitor. If the output voltage is not a relevant measure, for example, in current source applications, capacitors can even be left unconnected. A relay activated by the digital controller is present, allowing to connect/disconnect the board to external circuits. Fig. 4 shows a representation of a power board. A detailed schematic is provided in Fig. 6.

The boards can be arbitrarily interconnected to compose the desired conversion circuit. The power boards can be supplied by exploiting the dc-link board, discussed next.

### 2.2 DC-link board

**Fig. 5:**
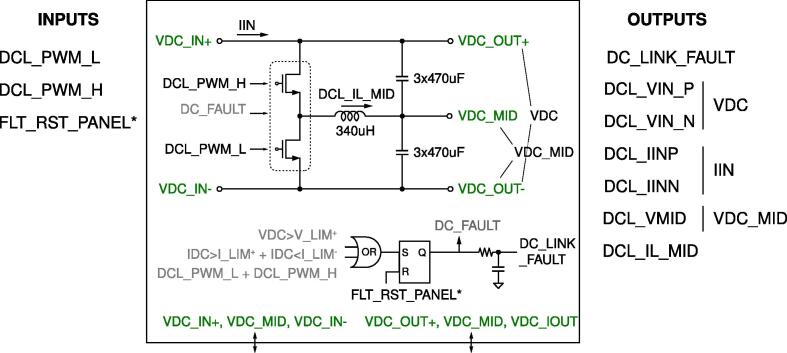
Schematic representation of the dc-link board. On the left, the input signals of the board, on the right, the output signals generated by the board. On the bottom, colored in green, the power lines made available at dedicated connectors. The complete schematic of the board is provided in Fig. 11, Fig. 12.

**Fig. 11:**
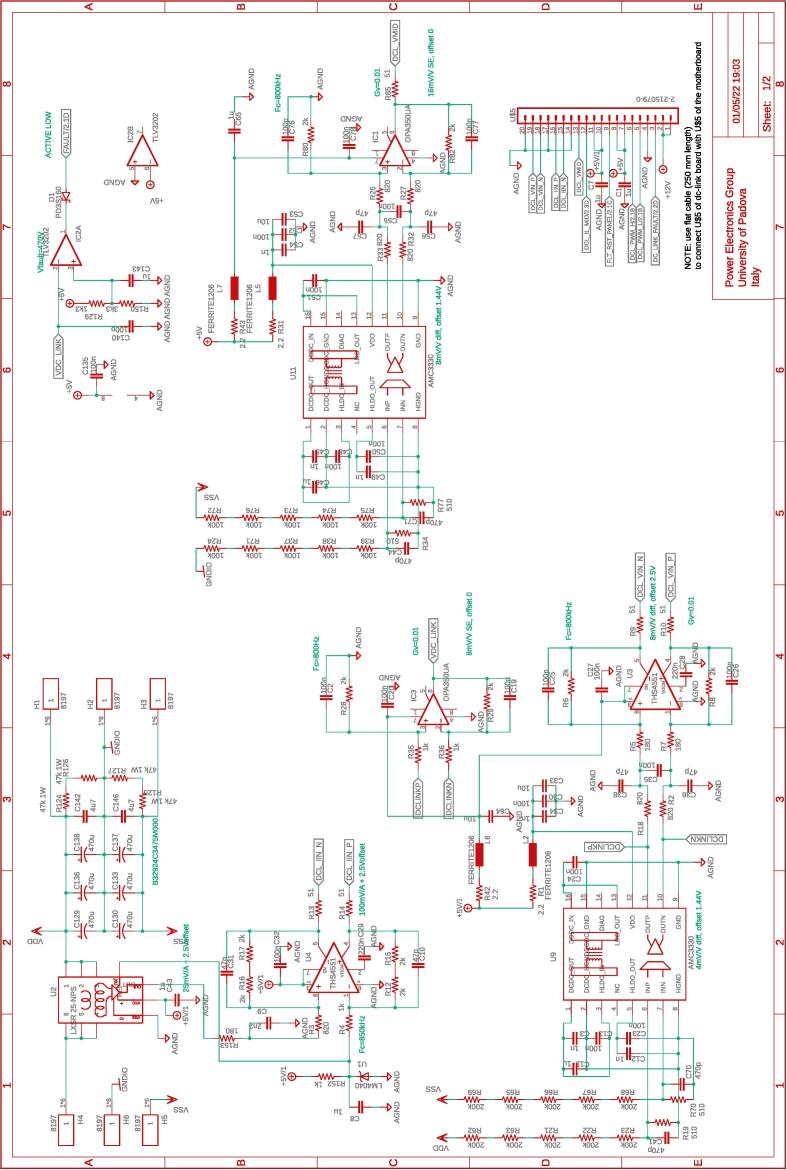
DC-link board hosting the dc-link capacitors, dc-link voltage sensor, split dc-link voltage sensor, input dc-link current sensor, overvoltage protection.

**Fig. 12:**
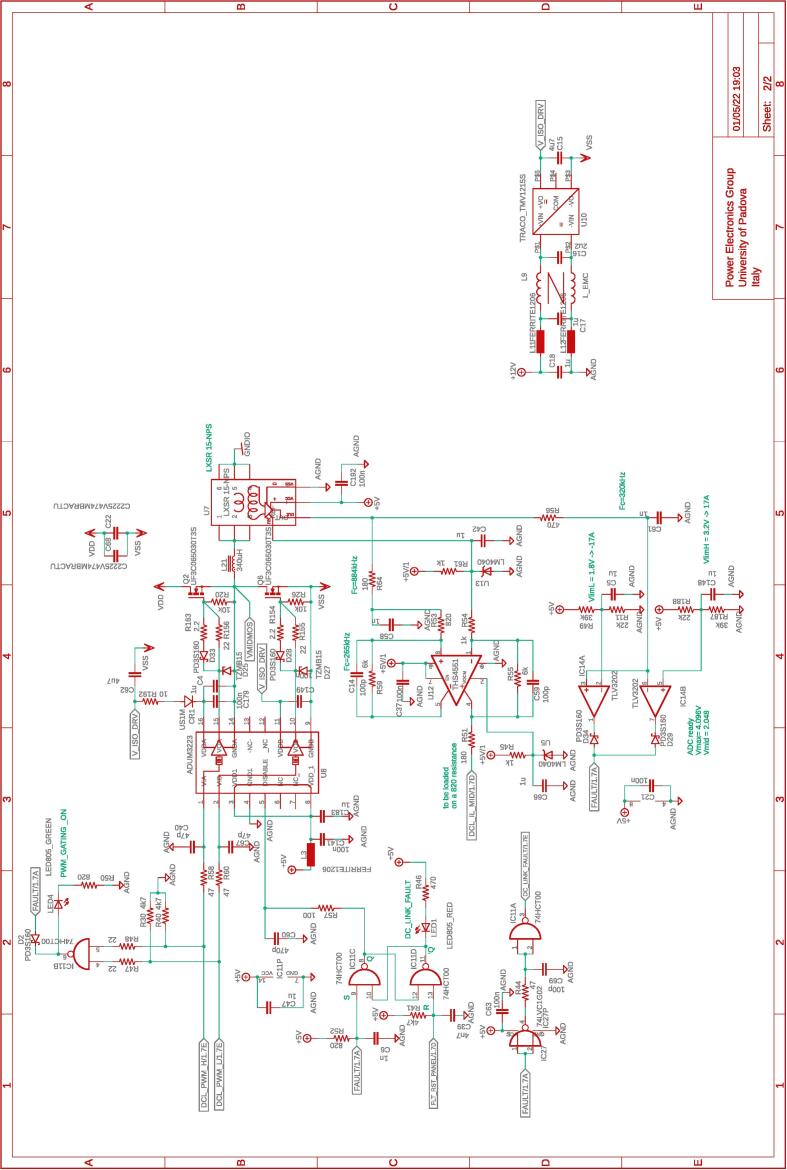
DC-link board hosting the power electronic circuits for active split dc-link control.

The dc-link board hosts a dc-link capacitor bank, visible in Fig. 11, useful in many conversion circuits. The bank is constituted by the series and parallel connection of six capacitors rated 450 V, 470 μF each, for an equivalent total capacitor of 700μF split in two series-connected blocks of 1.4mF with the central connection point made available. In addition, the central connection point is connected via a 340-μH inductor to a half-bridge, as shown in Fig. 12, which can be used for active control of the mid-point voltage. For this purpose, the inductor current and mid-point capacitor voltage are measured and brought to the digital controller as analog inputs. A schematic representation of the dc-link board is shown in Fig. 5.

### 2.3 Digital controller

The adopted digital controller in this project is the B-Board Pro by Imperix l.t.d [18]. Such a digital controller embeds a Xilinx Zynq system-on-chip, which integrates a couple of ARM processors with 1GHz clock frequency, and a Kintek-7 FPGA. A set of high-performance peripherals for converters control is also present, including analog-to-digital-converters (ADC), pulse-width modulated (PWM) digital outputs, general purpose input/output lines, communication ports, on board memory.

This controller has been chosen being it fully programmable by MATLAB/Simulink, besides the more classical programming language C/C++. By exploiting the integrated code generation features of MATLAB/Simulink, it is possible to program the digital controller connected to the proposed hardware and apply a rapid control prototyping approach. By RCP, the traditional development process toward experimental implementation—that typically consists of modeling, validation in simulation, code development, and deployment—is significantly lightened because code development is not required. This is beneficial for time to market in industrial environments, while, in academic and research environments, RCP helps to focus on concepts, theories, models, and algorithms bypassing the code development stage, which can instead be treated in other separate dedicated teaching modules.

### 2.4 Motherboard

**Fig. 8:**
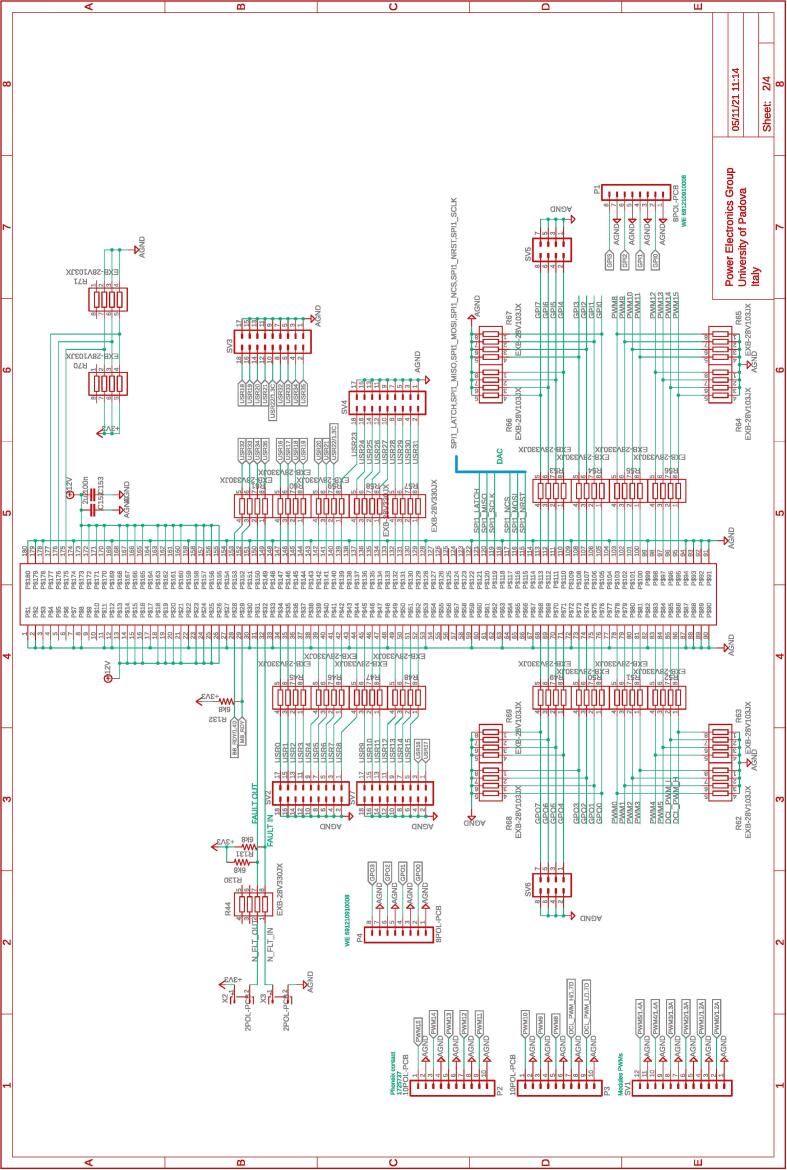
Motherboard hosting the digital controller. Circuit 2/4 related to physical connections with the digital controller (i.e., B-Board-PRO) and user-side screw-connectors to allow the availability of the general-purpose digital input/outputs lines.

**Fig. 9:**
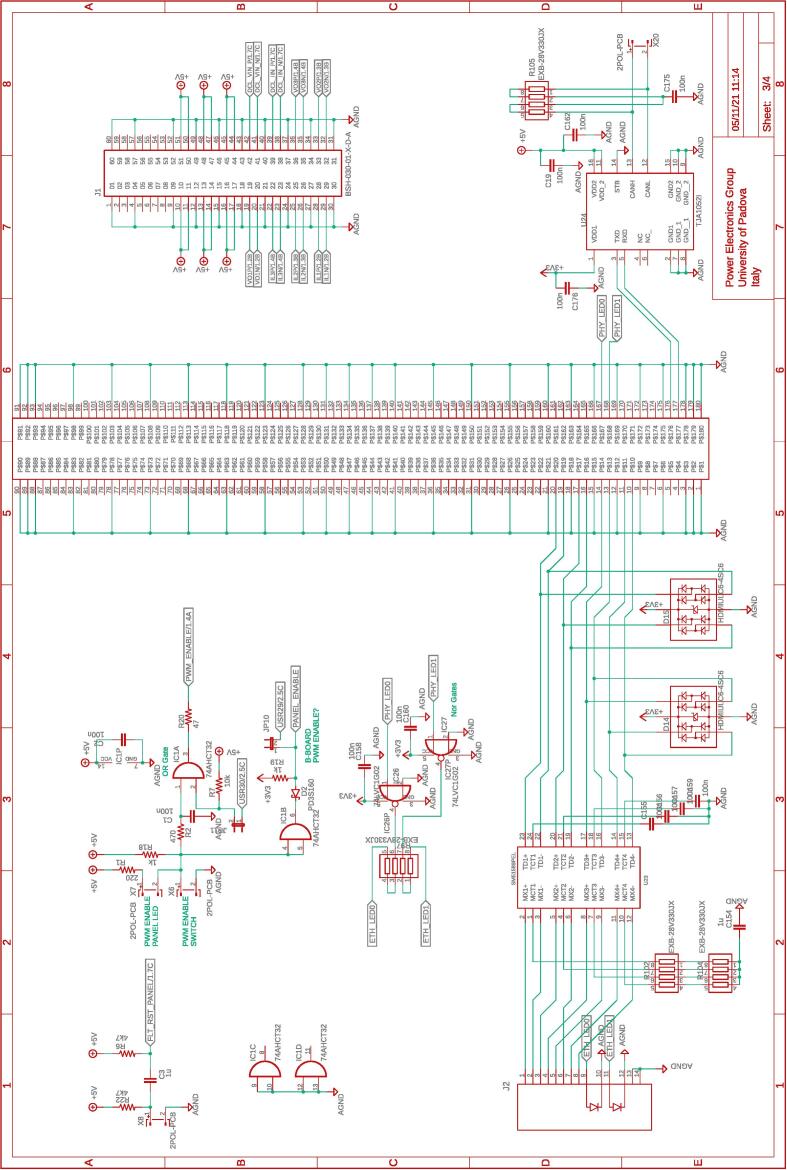
Motherboard hosting the digital controller. Circuit 3/4 related to the physical connection with the digital controller (i.e., B-Board-PRO) of the ethernet and CAN communication, the differential analog input channels to the digital controller, and the management of enable commands.

The several boards constituting the conversion and control system are interconnected via a dedicated board called motherboard herein. The motherboard presents sockets for installing the adopted digital controller Imperix B-Board Pro®, the three power boards, and the dc-link board. Flat cables are employed to connect the motherboard with the DC-link board and the power boards. The motherboard routes the digital and analog lines from the boards hosting the conversion circuits to the digital controller. The motherboard also embeds four digital-to-analog converters and four additional analog-to-digital converters that can be accessed via the digital controller. The complete schematic of the motherboard are provided in Fig. 7, Fig. 8, Fig. 9, Fig. 10.

### 2.5 On-board supply and fan-speed controller

An on-board power supply model MW IRM-90-12ST, with 12 V, 6.7 A output, is included in the prototype for supplying all the electronics circuits for analog signal conditioning, sensors, digital circuits, and the digital controller. The power supply is coupled with an electromagnetic interference (EMI) filter model Schaffner FN922S-3-06, a fuse, and an on/off switch.

**Fig. 13:**
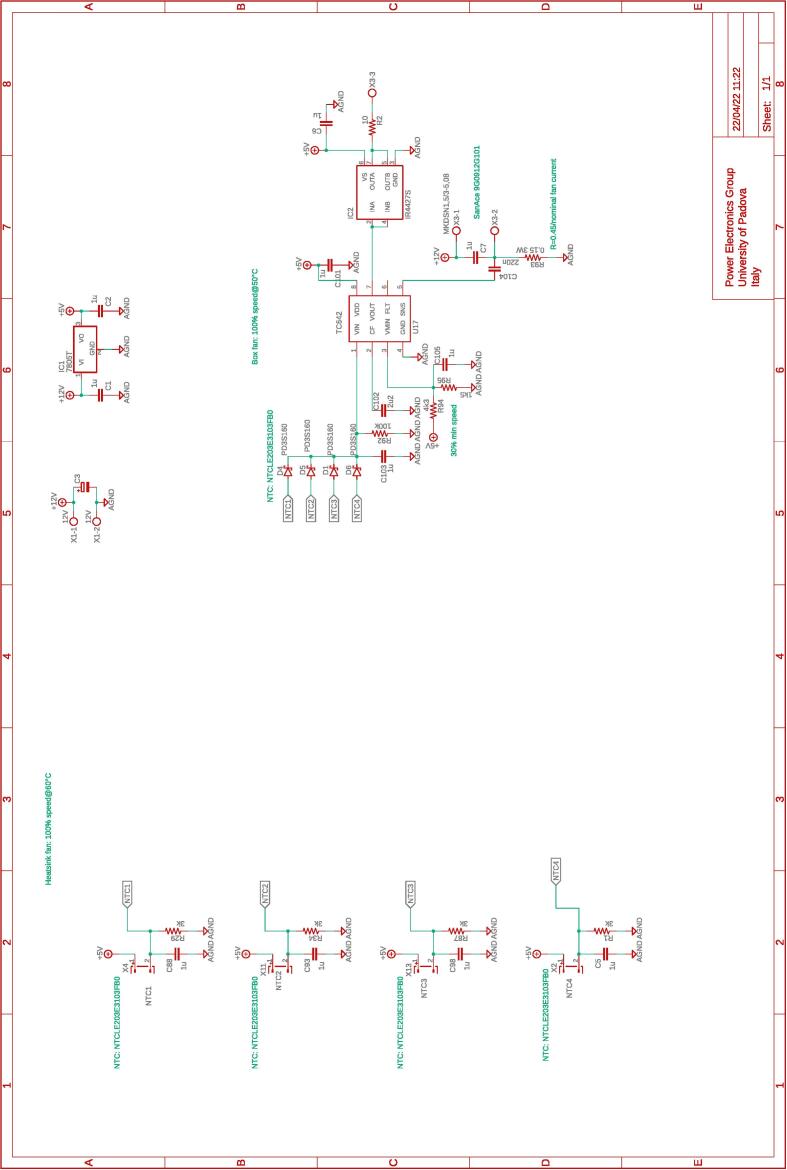
Board for speed control of fan coolers.

An auxiliary board is also present to regulate the speed of the fan coolers for the electronic devices of the power boards. The speed regulation is provided by the special function integrated circuit TC642 based on the maximum temperatures of the heatsinks in the three power boards and the dc-link board. The complete schematics of the boards are shown in Fig. 13.

### 2.6 Protection hierarchy

**Fig. 2:**
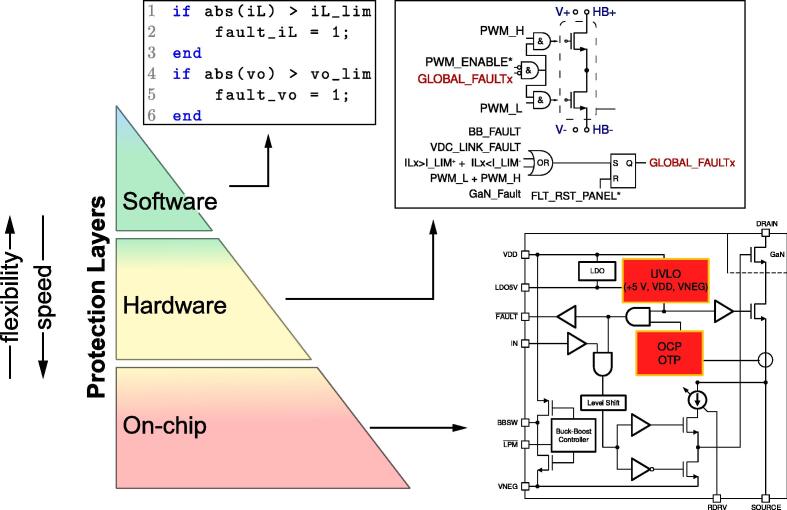
Schematic representation of the protection layers.

This system has been designed to be operated both by research professionals and by students. To minimize failures due to over-currents, over-voltages, or over-temperatures, a hierarchy of protection methods has been included. The protection provisions include on-chip protections, hardware on-board protections, and software protections, as represented in Fig. 2.

First, the GaN module embeds on-chip over-current, over-voltage, over-temperature, and under-voltage lock-lockout protection. Being integrated into the power devices, these protections are the fastest-acting ones and are expected to trigger during hazardous operating conditions that would otherwise destroy the electronic devices.

Then, other fault protection mechanisms are implemented on hardware in the electronic boards, both in the half-bridge power boards and in the dc-link board. Specifically, the half-bridge power boards host circuits that implement over-current and shoot-through protection of the power devices. In addition to over-current and shoot-though protections, the dc-link board also implements over-voltage protection that triggers when the dc-link voltage in the dc-link board transcends the maximum limit of 470 V. Only the over-current hardware protection of the half-bridge power boards can be adjusted by means of a couple of trimmers located on the motherboard. An active-low logical circuit manages the several fault signals such that, whenever at least one of the safety conditions is violated, an alarm signal is generated and latched, which disables the PWM modulations in the interested peripheral board. In order to re-enable the system, fault conditions must be cleared using the reset button, indicated in Fig. 1.

Finally, an additional safety layer can be implemented in software, that is, in the Simulink model representing the control code to be deployed in the digital controller (i.e., the B-Board Pro). A simple yet effective software protection is implemented in the firmware template provided with the manuscript. Software protections are typically slower and less accurate than hardware or on-chip protection. Still, they allow significant flexibility in defining adjustable thresholds or ad hoc protection schemes, which is valuable during the debugging phase and to cease operation in case undesired events occur that bring the system to operate far from the expected operating conditions.

**Fig. 3:**
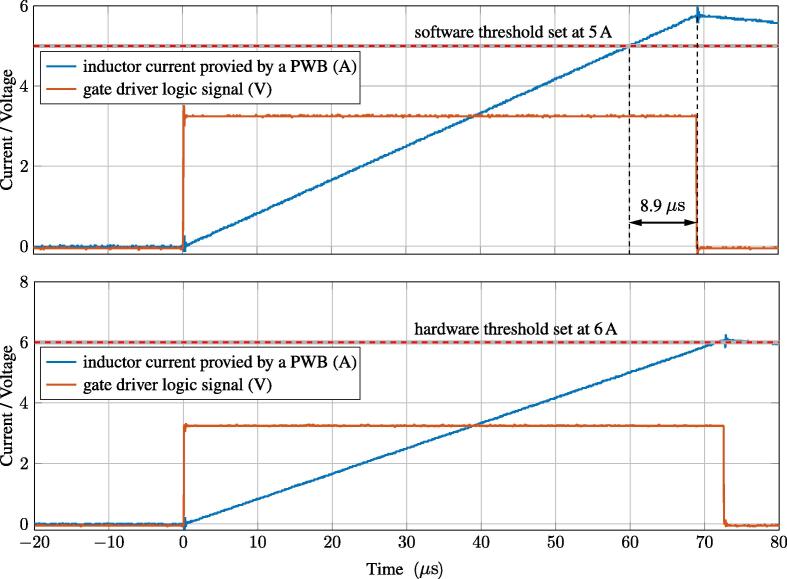
Demonstration of software and hardware current protections firing. Orange waveforms show the signal sent to the transistors drivers. Blue waveforms show the inductor current rising and then falling after transcending the set threshold. The plot on the top highlights that software protections, as typically occur, may present intervention delays of up to a sampling period, which often equals a switching cycle (i.e., 10μs in this case) and is commonly tolerable in practice.

**Fig. 7:**
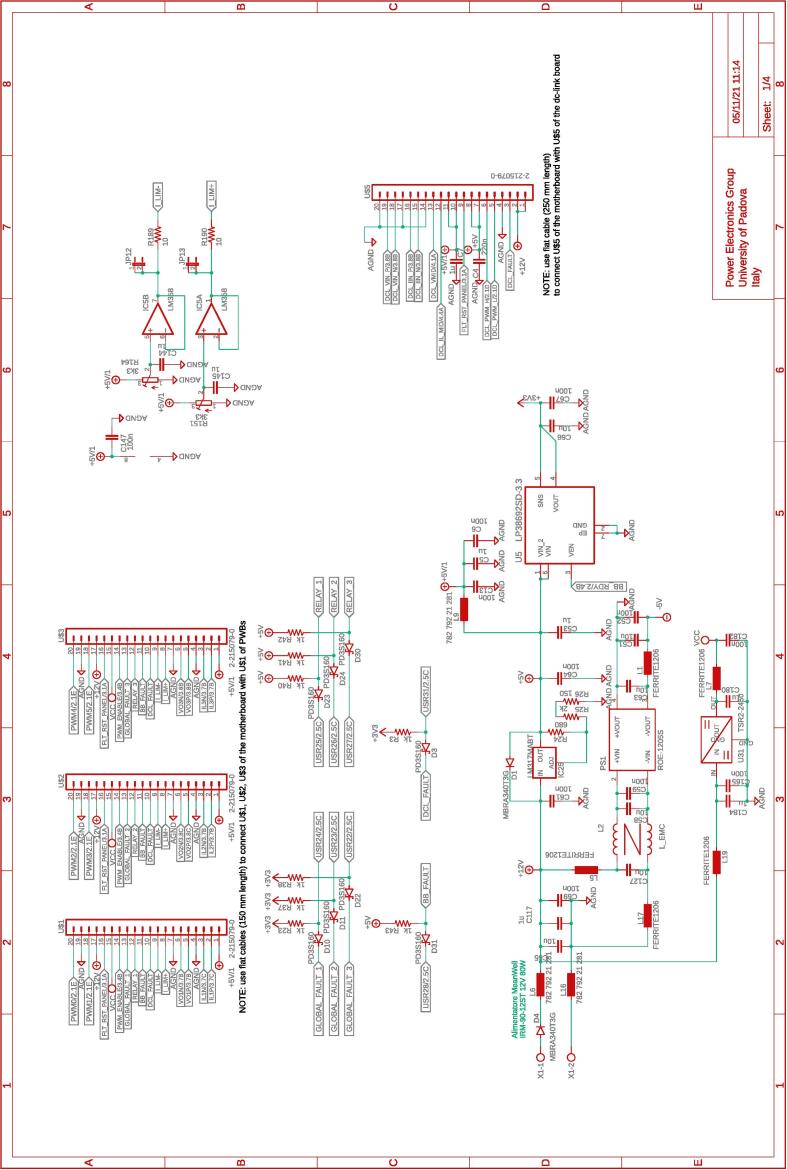
Motherboard hosting the digital controller. Circuit 1/4 related to the power supplies for digital controller and conditioning circuits.

Fig. 3 shows the intervention of the first two protection layers. The plot on the top shows the firing of the software protections, which presents the advantage of being easily set by the control panel, and the disadvantage of involving intervention delays of up to a switching cycle, that is, up to 10μs in the considered case. The plot on the bottom shows the firing of the hardware protections after an overcurrent event. In this case, the intervention delay is limited to the signal propagation delays through the gates of the digital circuits implementing the hardware protection scheme in Fig. 6, which amount to a few tens of nano-seconds. The overcurrent threshold can be set by the trimmers R164 and R151 in Fig. 7. Eventually, on-chip protections are integrated in the selected GaN half-bridge modules LMG3411EVM-018 and fire only in case of dangerous conditions. The characteristics and performances of on-chip protections are fully specified and shown in [19].

In the end, based on the highlighted features and structure of the converter hardware and control, the proposed system can be used, for example, for:

• implementation of power electronics circuits for teaching purposes;
• implementation of dc-dc switching power supplies;
• implementation of dc-ac switching power supplies;
• implementation of bidirectional dc-dc and dc-ac active loads;
• study of digital controllers for dc-ac power electronics inverters connected to the ac-grid;
• generation of ac perturbation signals for small-signal characterizations;
• study of the operation of parallel-connected converters.

Application examples showing and validating the proposed system are discussed in Sect. 7.

## 3 Design files

All the design files are freely available on the on-line repository [4].

**Table:** 

**Design filename**	**File type**	**Open source license**	**Location of the file**

dc_link_BOM.xlsx	BOM of Fig. 11, Fig. 12	CERN-OHL-P	Hardware/dc_link
dc_link_v0.brd	Eagle PCB layout of Fig. 11, Fig. 12	CERN-OHL-P	Hardware/dc_link
dc_link_v0.sch	Eagle PCB schematic of Fig. 11, Fig. 12	CERN-OHL-P	Hardware/dc_link
fanBoard_bom.xlsx	BOM of Fig. 13	CERN-OHL-P	Hardware/fanboard
fanBoard_simple.brd	Eagle PCB layout of Fig. 13	CERN-OHL-P	Hardware/fanboard
fanBoard_simple.sch	Eagle PCB schematic of Fig. 13	CERN-OHL-P	Hardware/fanboard
motherboard_BOM.xlsx	BOM of Fig. 13	CERN-OHL-P	Hardware/fanboard
motherboard_v0.brd	Eagle PCB layout of Fig. 7, Fig. 8, Fig. 9, Fig. 10	CERN-OHL-P	Hardware/motherboard
motherboard_v0.sch	Eagle PCB schematic of Fig. 7, Fig. 8, Fig. 9, Fig. 10	CERN-OHL-P	Hardware/motherboard
hb_power_board_BOM.xlsx	BOM of Fig. 6	CERN-OHL-P	Hardware/powerboard
hb_power_board_v0.brd	Eagle PCB layout of Fig. 6	CERN-OHL-P	Hardware/powerboard
hb_power_board_v0.sch	Eagle PCB schematic of Fig. 6	CERN-OHL-P	Hardware/powerboard
init_template.m	MATLAB initialization file	CERN-OHL-P	Software/powerboard
model_template.slx	Simulink Template	CERN-OHL-P	Software

## 4 Bill of materials summary

The bills of materials for implementing the system shown in Fig. 1 are available on the repository [4].

## 5 Build instructions

**Fig. 10:**
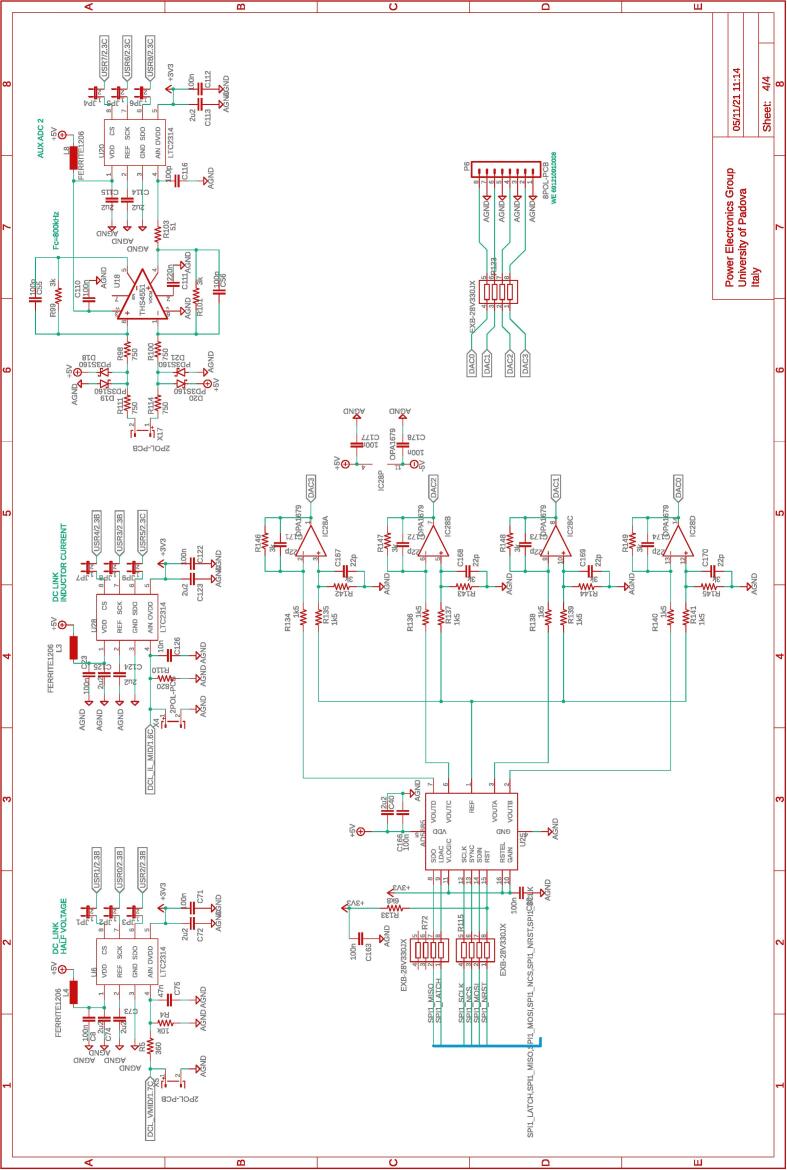
Motherboard hosting the digital controller. Circuit 4/4 related to the auxiliary ADCs, DACs, and related analog-signal conditioning.
• the dc-link board, whose schematics are displayed in Fig. 11, Fig. 12.

Most of the building effort is related to the assembly of the printed-circuit-boards (PCBs) of the boards from Fig. 6, Fig. 7, Fig. 8, Fig. 9, Fig. 10, Fig. 11, Fig. 12, Fig. 13. Regarding what is presented herein, these boards have been fabricated externally, relying on a PCB prototyping and assembly company (e.g., Eurocircuits, PCBWay). All the design files to be submitted to such companies are available in the repository [4]. Instead, components assembly has been performed manually in the authors’ laboratories by standard PCB assembly methods. In this regard, only basic knowledge of PCB manufacturing is required (see, e.g., [20] for reference) once the design files provided in the repository are available.

**Fig. 14:**
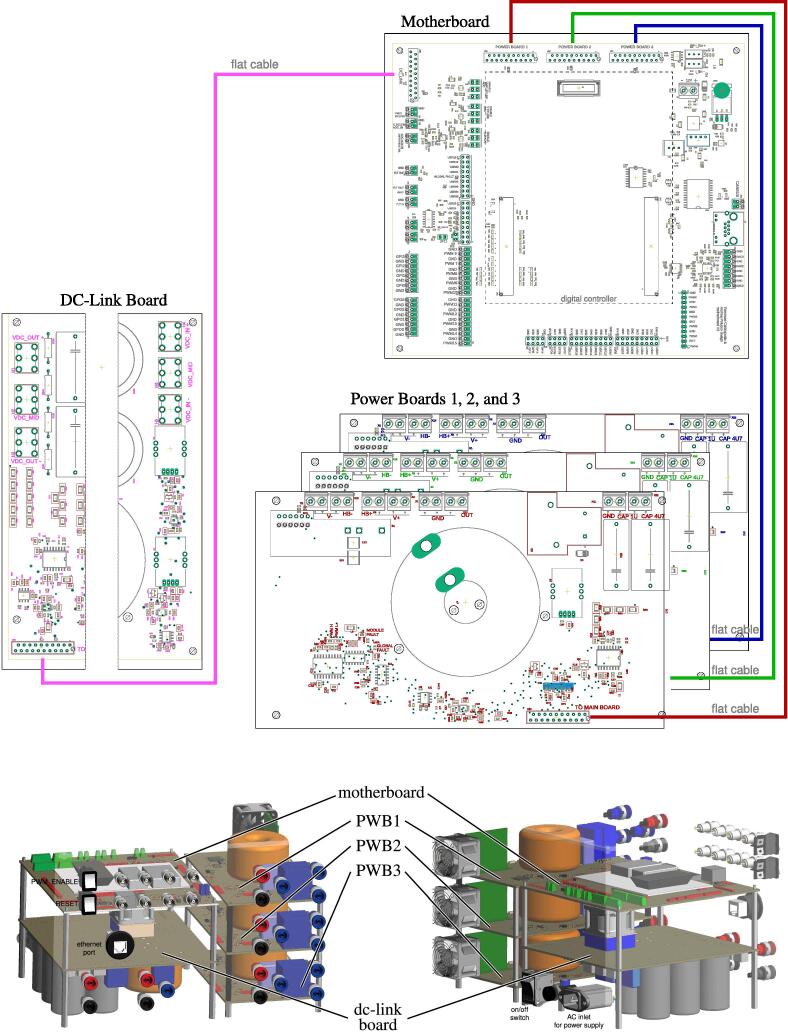
Interconnection of the boards shown on the top part of the figure with a possible disposition and arrangement of the boards on the bottom.

Fig. 14 shows the connections among the main boards and displays a possible layout. A different positioning of the boards may be possible too. Specifically, the figure displays:

• the power boards, whose schematics are displayed in Fig. 6
• the motherboard, whose schematics are displayed in Fig. 7, Fig. 8, Fig. 9, Fig. 10 and

**Fig. 17:**
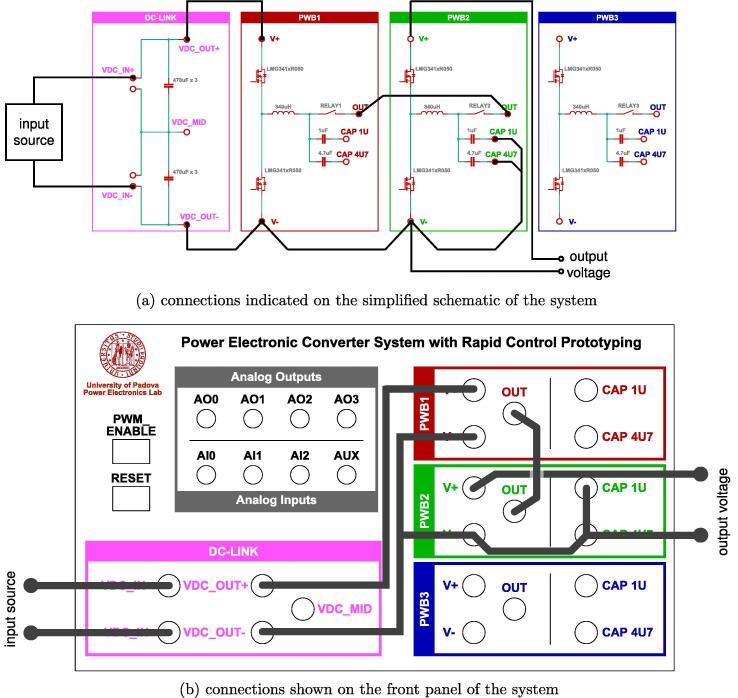
Connections required for the implementation of the application represented in Fig. 16.

**Fig. 22:**
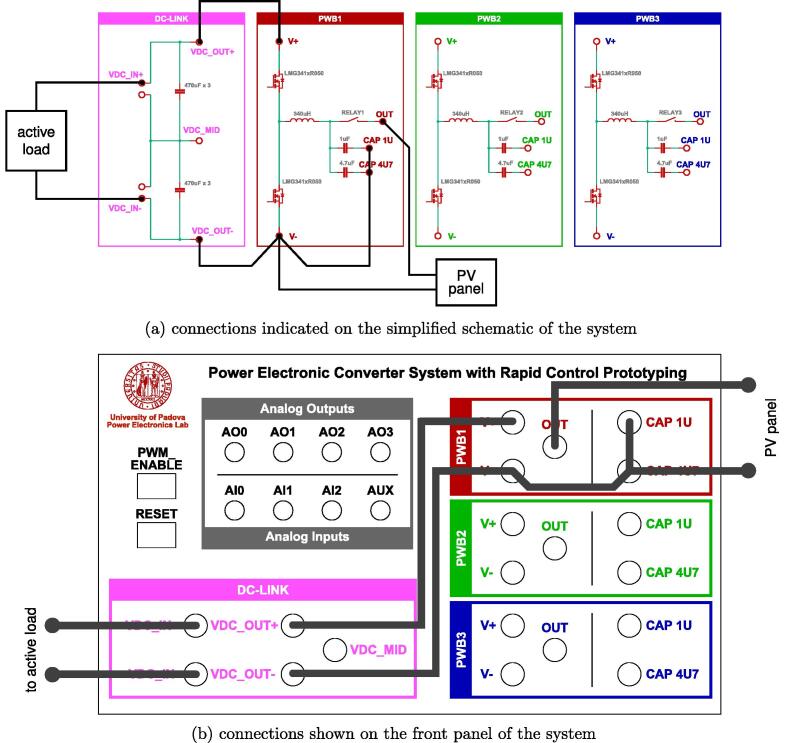
Connections required for the implementation of the application represented in Fig. 21.

The fan control board in Fig. 13 is not shown, being it an independent auxiliary board to be used if system cooling is required. Notably, the labels appearing on the principle schematics in Fig. 4, Fig. 5 of the power lines made available at dedicated connectors related to the dc-link board and the half-bridge power-boards correspond to the labels appearing on the circuit schematics in Fig. 6, Fig. 7, Fig. 8, Fig. 9, Fig. 10, Fig. 11, Fig. 12, and on the PCB realizations available on the shared repository [4] and displayed in Fig. 14. In addition, such labels are reported in the application examples displayed in Fig. 17 and in Fig. 22.

**Fig. 15:**
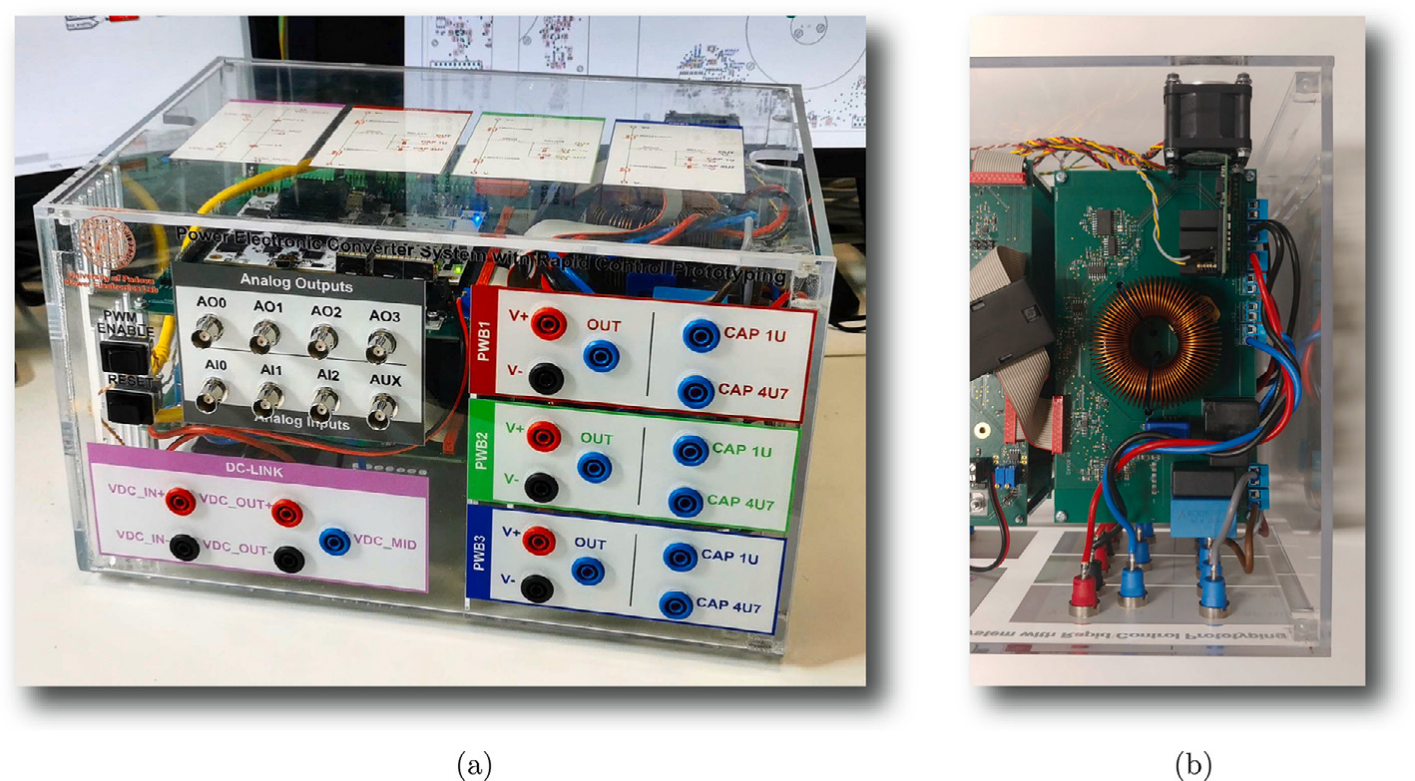
Implemented flexible power electronic converter system with rapid control prototyping, (a) overall aspect of the system, (b) implementation of the half-bridge section with power boards (PWBs).

A photo of the physical realization of the proposed system is provided in Fig. 15. Fig. 15a shows the appearance of the final overall implementation based on the design files indicated in Sect. 3 and layout proposed in Fig. 14. Fig. 15b displays the implemented power board (i.e., PWB1 according to the layout in Fig. 14). Interconnections indicated in Fig. 14, top, among boards PWB1-PWB3 in Fig. 6 and dc-link in Fig. 11 with the motherboard in Fig. 7 are performed using flat cables (i.e., ribbon cables), included in the BOM available in [4] and noted in the schematics of Fig. 6, Fig. 7, Fig. 8, Fig. 9, Fig. 10, Fig. 11. Ferrite clamp-on cores are also used (e.g., model Fair-Rite 0443166651) and recommended for wounding the ribbon cables to reduce EMI issues.

## 6 Operation instructions

For what concerns the coding of the system firmware, it can be generated by using Simulink® environment employing Imperix ACG SDK® blockset, which can be freely downloaded on-line [18]. Imperix blockset includes blocks to read ADC channels and drive PWM channels. A template file is available in the repository [4], where PWM and ADC channels are labeled respecting the same nomenclature in Fig. 6, Fig. 5. Once the code is deployed on the control board, the operation can be monitored and managed using Imperix Cockpit®, which automatically starts after the code is generated. In order to correctly implement a power converter:

1. Prepare in Simulink a control algorithm to be tested on the prototype and, if necessary, a model of the controlled system; this can be done, for example, by using the template provided in the repository [4].
2. Debug and eventually deploy the controller on the final digital controller. To this end, an RCP development procedure consisting of simulation and deployment, without additional intermediate steps, within the same development environment can be applied.
3. Setup the needed interconnections among the various boards based on which system is intended for implementation.
4. Build the code to be deployed in the control board; Imperix Cockpit® should start and show up.
5. Check the IP and connect to the correct control board.
6. Setup Cockpit interface (variables to be monitored, scopes) and eventually modify control variables that need to be set up.
7. Reset the prototype by pressing the reset button on the front panel.
8. Enable PWM in order to start the operation of the system.

## 7 Validation and characterization

Three examples are reported in the following to show real-world applications of the proposed rapid prototyping system. The first example considers the implementation of a four-switches buck-boost power converter, which allows reporting the effective operation of the developed system while referring to a circuit configuration of practical interest. The second example is a teaching activity relevant to undergraduate or graduate courses covering aspects of electronics for energy. The third example reports a research activity that exploits the proposed system to collect the experimental results needed for validating an artificial intelligence approach in the field of power electronics. This last example shows the application of the proposed system for implementing a test setup relevant to a current research field.

### 7.1 Application Example #1: four-switches buck-boost converter

In this example, a four-switches buck-boost converter has been implemented. This converter is widely used in several applications, including photovoltaic energy harvesting [21], dc low-voltage microgrids [22], and telecommunications power supplies [23].

**Fig. 16:**
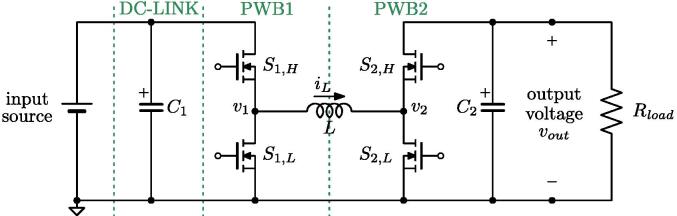
Four-switches buck-boost topology of the application example in Sect. 7.1.

The topology is shown in Fig. 16. The corresponding connections required for its implementation on the proposed flexible power electronic converter system are illustrated in Fig. 17, considering PWB1 and PWB2. Fig. 17a indicates the connections on the simplified schematic of the system, while Fig. 17b displays the corresponding connections on the front panel. This configuration is used here to validate the setup by showing the main waveforms of the circuit during operation with output resistive load of 50 Ω and input voltage and output voltage of 60 V and 30 V, respectively. Finally, $C_{1}$ is the equivalent capacitor in the dc-link board and PWB1, $C_{2}$ is the equivalent capacitance in PWB2.

**Fig. 18:**
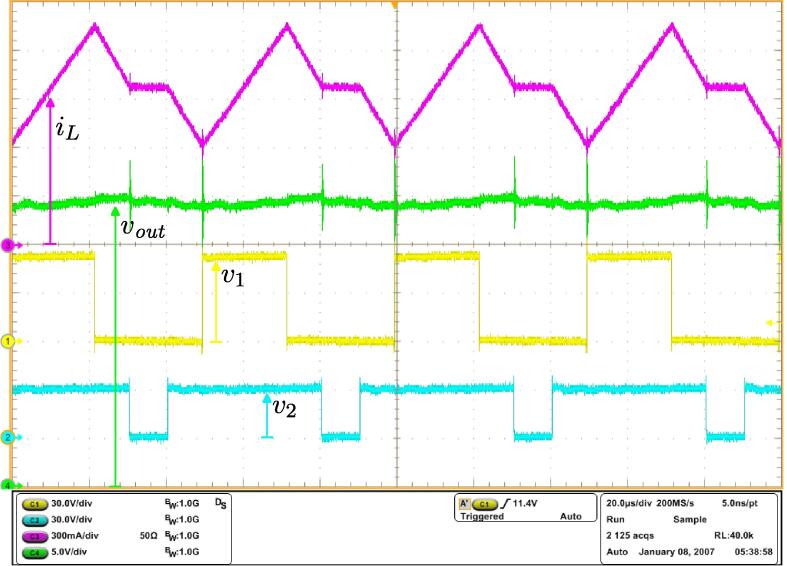
Experimental waveforms for the four-switches buck-boost power converter in Fig. 16. From top to bottom: inductor current, output voltage, and voltages at both switching nodes.

**Fig. 19:**
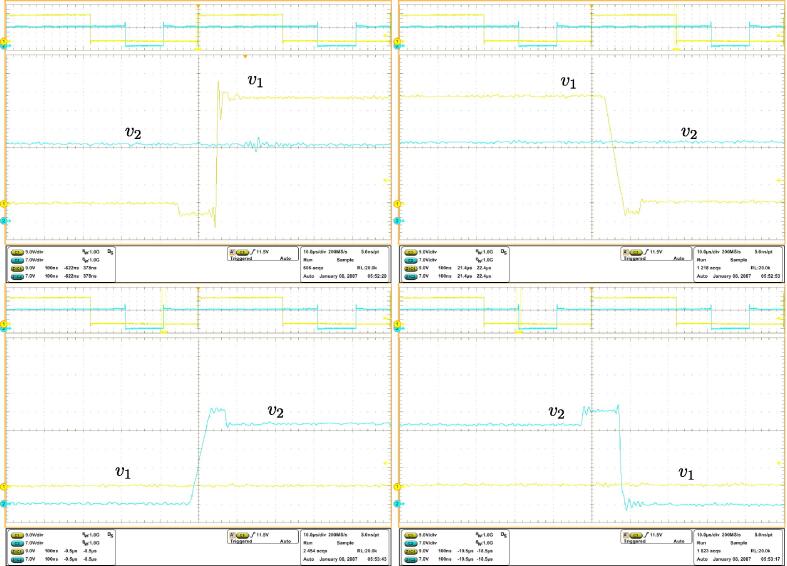
Voltage waveforms acquired at both switching nodes of the circuit in Fig. 16. The rising and falling edges of the commutating voltages appear clean, as expected from the technical sheets of the adopted electronic switches [19].

Various waveforms are reported: Fig. 18 shows, from top to bottom, the inductor current, the output voltage, and the voltages at the switching node of PWB1 and PWB2. Fig. 19 shows clean transitions during switching intervals at the switching nodes, both for the rising and the falling edges. As an additional detail, minor from the perspective of the proposed solution but a well-known peculiarity of GaN FETs wide bandgap devices (in this case, the TI LMG341xR050), it is possible that the switching nodes voltages present visible variations when third-quadrant mode occurs for the devices around the voltage transitions. This is due to the significant source-drain voltage of the GaN FET devices in this mode, which amounts to about 4 V in this case.^1^

**Fig. 20:**
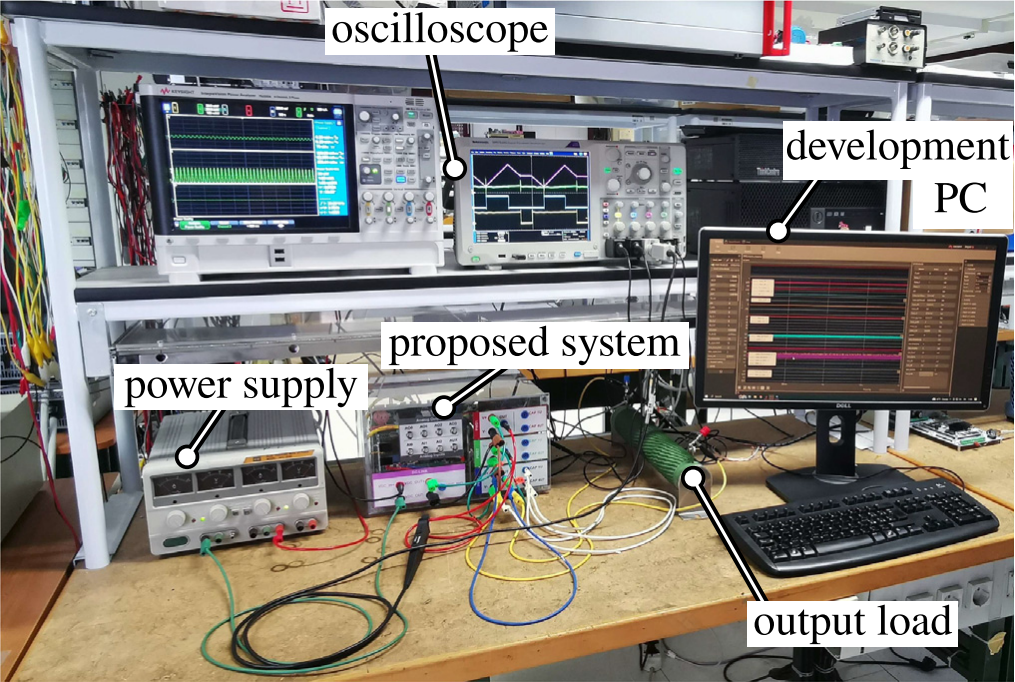
Photo of the hardware setup related to Sect. 7.1.

A picture of the hardware setup is reported in Fig. 20.

### 7.2 Application Example #2: photovoltaic energy harvesting

Photovoltaic (PV) energy harvesting is a timely application commonly included in electrical and electronics engineering university courses. Topics like maximum power point extraction for renewable sources like PV, dc-dc converter control for maximum power point extraction, renewable sources electrical interfaces, and modeling of the primary renewable source are discussed in several recent textbooks relevant to the field (see, e.g., [24], [25]). This paragraph demonstrates the use of the proposed system for a teaching laboratory activity on this topic.

For this application, the system proposed in this article removes the need to develop a dedicated system (i.e., selection of components, design and realization of the PCB, debugging), thus limiting the related expenses, and the trainees can be exposed to a hands-on activity by interconnecting the converters and performing the measurements themselves. Moreover, the rapid prototyping framework in the system allows students to implement and test the control algorithm on their own.

**Fig. 21:**
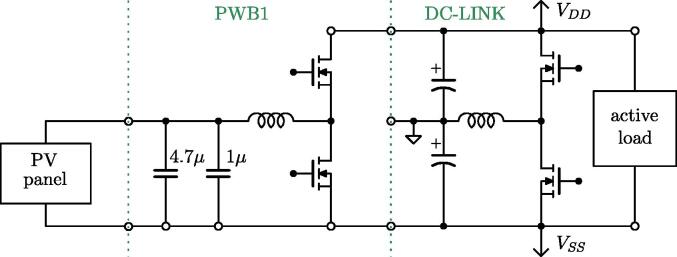
Schematic of the setup for solar energy harvesting in Sect. 7.2.

In this example, a 230W photovoltaic panel is connected to a half-bridge power board, implementing a boost converter. The output of the boost converter is connected to an active load set in voltage control mode (i.e., the load voltage is regulated to a user-defined value). Being the power board a half-bridge circuit, to implement a boost converter, the output of the power board is considered as the input of the boost, while the original input of the board is taken as the boost output. The boost converter constituted by a power board has limited output capacitance (see Fig. 6), then it is connected to the active load through the dc-link board, whose capacitor bank plays in this way the role of output capacitor. The conceptual schematic of the setup is shown in Fig. 21. The corresponding connections on the prototype are displayed in Fig. 22, where Fig. 22a indicates the connections on the simplified schematic of the system, while Fig. 22b displays the corresponding connections on the front panel.

**Fig. 23:**
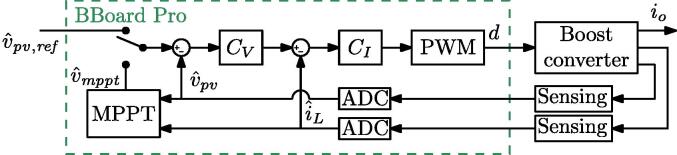
Control scheme employed for the solar energy harvesting experiment.

The controlled variable of the boost converter is the input voltage, which corresponds to the output voltage of the power board. Since the boost output voltage is fixed thanks to the regulation provided by the active load set in voltage control mode, it is possible to regulate the input voltage of the converter by appropriately setting the duty-cycle of the converter, which is done by a dedicated regulator implemented in the digital controller. In this case, the control is implemented using two loops: an outer voltage loop sets the reference value for the inner current loop, which defines the duty-cycle value to be applied to the converter. The reference voltage of the loop, which corresponds to the photovoltaic-panel voltage, can be set to a user-defined value or through a maximum power-point tracking (MPPT) algorithm that searches for the voltage that maximizes the energy extraction. A block scheme of the control system is shown in Fig. 23.

**Fig. 24:**
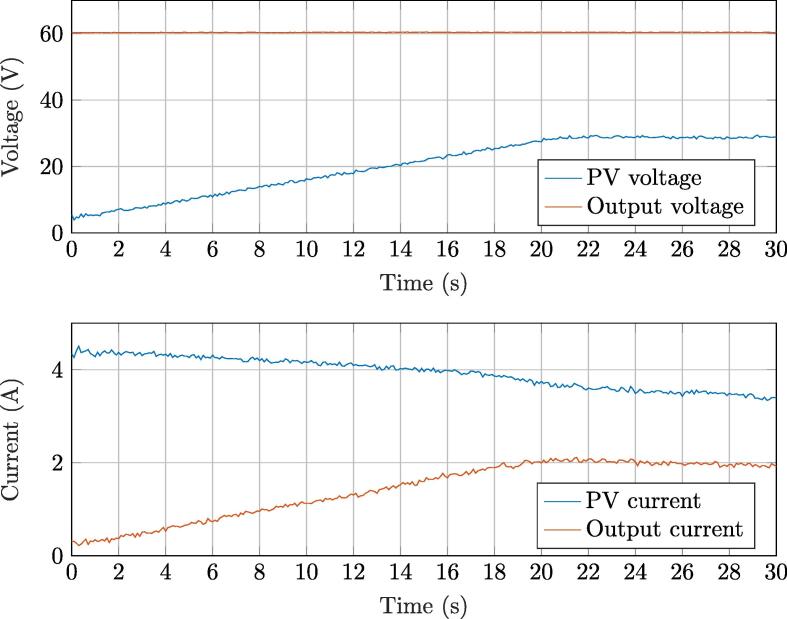
MPPT algorithm searching the optimal voltage maximizing solar power production.

Fig. 24 shows the resulting waveforms from representative test. Initially, at t=0s, the PV panel voltage is kept fixed a 5V, while the boost output voltage is regulated at 60V by the active load. The PV panel outputs about 4.2A. At t=1s, the MPPT algorithm is activated, then, the PV voltage gradually increases until about t≃19s, instant at which a maximum power point is detected. By looking at the output current, it is possible to notice that solar generation of about 120W was obtained at the time of the test. Noticeably, specific choices in the implementation (e.g., the considered initial condition of 5 V for the MPPT control test) have only a demonstrative value: different initial conditions may be considered in the implementation at hand, according to the actual application and aims of the user.

### 7.3 Application Example #3: dataset collection for AI training

**Fig. 25:**
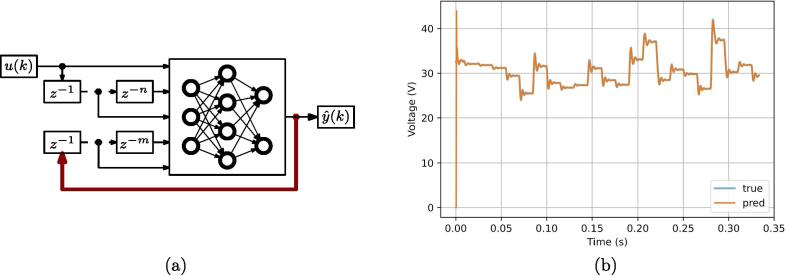
Sample of results related to application example #2 in Sect. 7.3. (a) Structure of a NARX artificial neural network; (b) output voltage predicted by the AI approach (orange) versus experimental measurements (blue) obtained by the proposed rapid prototyping system. Additional results reported in [26].

In this example, a method based on artificial intelligence (AI) for modeling electronic power converters is considered and demonstrated using the prototyping setup presented in this paper. This method was proposed in [26]. An essential aspect of AI-based methodologies is the generation of the dataset to be used for the training of the AI-based models. Various dc-dc topologies should be considered to validate the modeling approaches with different configurations, for which purpose the proposed flexible system reveals particularly useful. For each one, datasets corresponding to several operating conditions should be generated and collected for the optimal training of the AI-based model. The general-purpose system proposed herein allows to develop various conversion circuits (i.e., topologies) without requiring dedicated circuit design and PCB development for each converter to be tested. By employing this system as described in [26], it has been possible to efficiently implement the topologies to be investigated and quickly deploy the system setup for validation. With the generated dataset, a NARX neural network has been trained to forecast the current and voltage behavior of the system, as represented in Fig. 25, thus modeling its dynamic behavior. Additional details can be found in [26].

## 8 Conclusions

A flexible power electronics energy conversion system has been presented in this paper. The system comprises a set of electronics boards devised to allow maximum flexibility for the rapid prototyping of conversion circuits useful in laboratory setups and teaching purposes. In the current energy scenario, such systems find countless applications, which are expected to increase further in the coming years. Examples include the development and study of buck, boost, and three-phase converters for renewable energy sources interface and integration, switching-mode power supplies, motor drives, etc. The work describes in detail the hardware structure of the system, includes the schematics and design files required to reproduce the related hardware, and discusses a demonstration of the use of the setup.

## Declaration of Competing Interest

The authors declare that they have no known competing financial interests or personal relationships that could have appeared to influence the work reported in this paper.

## Footnotes

1 Refer to [19] for additional details considering the components used herein.

## References

[1] Syndem, LLC (2022). [link]. URL: http://www.syndem.com/.

[2] Zhong Q.-C., Wang Y., Dong Y., Ren B., Amin M. (2020). Go Real: Power Electronics From Simulations to Experiments in Hours: Versatile Experimental Tool for Next Generation Engineers. IEEE Power Electron. Mag..

[3] Taraz Technologies (2022). [link]. URL: https://www.taraztechnologies.com/pelab/.

[4] T. Caldognetto, A. Petucco, A. Lauri, P. Mattavelli, Design files of the project “power electronic converter system with rapid control prototyping” (2022). URL: https://doi.org/10.17632/8tdd662jgp.2.

[5] Singh R., Paniyil P., Zhang Z. (2022). Transformative Role of Power Electronics: In solving climate emergency. IEEE Power Electron. Mag..

[6] Abb H. (2021).

[7] H. Chraye, Power electronics, a key technology for the renewable energy system integration, in: 2021 23rd European Conference on Power Electronics and Applications (EPE’21 ECCE Europe), 2021, pp. P.1–P.2. doi:10.23919/EPE21ECCEEurope50061.2021.9570548.

[8] Lehman B., Chung H.S.-H., Chen Y.-M., Li Y. (2019). Editorial 2019: Entering a New Era. IEEE Trans. Power Electron..

[9] S. Bonho, R. Pizzio, F.A.B. Batista, C.A. Petry, Teaching power electronics with engineering interdisciplinary projects, in: 2015 IEEE 13th Brazilian Power Electronics Conference and 1st Southern Power Electronics Conference (COBEP/SPEC), 2015, pp. 1–6. doi:10.1109/COBEP.2015.7420208.

[10] Mohan N., Undeland T., Robbins W. (2003). https://books.google.it/books?id=oxR8vB2XjgIC.

[11] Choi S., Saeedifard M. (2012). An Educational Laboratory for Digital Control and Rapid Prototyping of Power Electronic Circuits. IEEE Trans. Educ..

[12] Soomro J.B., Chachar F.A., Munir H.M., Ahmed Ansari J., Zalhaf A.S., Alqarni M., Alamri B. (2022). Efficient Hardware-in-the-Loop and Digital Control Techniques for Power Electronics Teaching. Sustainability.

[13] Mahmoudi H., Aleenejad M., Ahmadi R. (2018). Reconfigurable rapid prototyping platform for power electronic circuits and systems for research and educational purposes. IET Power Electron..

[14] Lamb J., Singh A., Mirafzal B. (2016). Rapid Implementation of Solid-State Based Converters in Power Engineering Laboratories. IEEE Trans. Power Syst..

[15] N. Kim, C. Roy, R. Cox, B. Parkhideh, A plug and play power electronics education board for hands-on learning of power converters incorporating wbg semiconductor, in: 2019 10th International Conference on Power Electronics and ECCE Asia (ICPE 2019 – ECCE Asia), 2019, pp. 2807–2813. doi:10.23919/ICPE2019-ECCEAsia42246.2019.8796999.

[16] Roy C., Kim N., Cox R., Parkhideh B. (2019). Development of a power electronics teaching lab incorporating wbg semiconductors with plug and play modular hardware and advanced curriculum. 2019 IEEE Energy Conversion Congress and Exposition (ECCE).

[17] Morais M.K. (2020). Teaching Power Electronics with the Aid of Open Source Simulation Tool eSim. 2020 IEEE Bombay Section Signature Conference (IBSSC).

[18] Imperix l.t.d., Inverter control board imperix b-board pro (2022). URL: https://imperix.com/products/control/inverter-control-board/.

[19] Texas Instruments, LMG341xR050 600-V 50-mIntegrated GaN Fet Power Stage With Overcurrent Protection datasheet (Rev. B), snosd81b Edition (Jan. 2020).

[20] Coombs C., Holden H. (2016).

[21] M. Orellana, S. Petibon, B. Estibals, C. Alonso, Four switch buck-boost converter for photovoltaic dc-dc power applications, in: IECON 2010–36th Annual Conference on IEEE Industrial Electronics Society, 2010, pp. 469–474. doi:10.1109/IECON.2010.5674983.

[22] M. Schulz, N. Schleippmann, K. Gosses, B. Wunder, M. März, Four switch buck/boost converter for dc microgrid applications, in: 2020 22nd European Conference on Power Electronics and Applications (EPE’20 ECCE Europe), 2020, pp. 1–10. doi:10.23919/EPE20ECCEEurope43536.2020.9215754.

[23] Ren X., Tang Z., Ruan X., Wei J., Hua G. (2008). Four switch buck-boost converter for telecom dc-dc power supply applications. 2008 Twenty-Third Annual IEEE Applied Power Electronics Conference and Exposition.

[24] Li Y., Nejabatkhah F., Tian H. (2022). https://books.google.it/books?id=-riAEAAAQBAJ.

[25] Keyhani A. (2019). https://books.google.it/books?id=5UCdDwAAQBAJ.

[26] A. Zilio, D. Biadene, T. Caldognetto, P. Mattavelli, Modelling Non-Linearities of Power Electronic Converters using Artificial Neural Networks (11 2022). doi:10.36227/techrxiv.21399519.v1. URL: https://www.techrxiv.org/articles/preprint/Modelling_Non-Linearities_of_Power_Electronic_Converters_using_Artificial_Neural_Networks/21399519.

